# Effect of combined chlorogenic acid and chitosan coating on antioxidant, antimicrobial, and sensory properties of snakehead fish in cold storage

**DOI:** 10.1002/fsn3.1378

**Published:** 2020-01-08

**Authors:** Xiaohuang Cao, Md. Nahidul Islam, Bimal Chitrakar, Zhenhua Duan, Wanxiu Xu, Saiyi Zhong

**Affiliations:** ^1^ College of Food Science and Technology Guangdong Provincial Key Labotatory of Aquatic Product Processing and Safety Guangdong Ocean University Zhanjiang China; ^2^ Department of Food Science Aarhus University Aarslev Denmark; ^3^ Laboration of Food Science and Technology Jiangnan University Jiangsu China; ^4^ Bioprocess Engineering Hezhou University Hezhou China; ^5^ Zhejiang Normal University Jinhua China; ^6^ Collaborative Innovation Center of Seafood Deep Processing Dalian Polytechnic University Dalian China

**Keywords:** coating, cold storage, microbial load, oxidization, quality, snakehead fish

## Abstract

Degradation of meat quality has always been a burning issue in fish preservation. To maintain the quality, a novel combination of chlorogenic acid (CGA) and chitosan (CS) coating was applied to snakehead fish fillets. Fish fillets were soaked into 2% chitosan (2CS), 0.2% CGA in 2% chitosan (0.2CGA/2CS), 0.5% CGA in 2% chitosan (0.5CGA/2CS), or 1.0% CGA in 2% chitosan (1.0CGA/2CS) solution; and then, coated samples were vacuum‐packaged and stored at 2 ± 0.5°C. pH values, color values, microbial loads, hardness, sensory qualities, and oxidization of lipids and proteins of stored fish fillets were investigated for 5 months. Antimicrobial activity was found to be nonsignificant (*p* ≤ .05) among different coated fish fillets, while color, antioxidant, and pH values were significantly (*p* ≤ .05) different. Lipid oxidation and protein oxidation were found to be inhibited in 2CS‐, 0.5CGA/2CS‐ and 1.0CGA/2CS‐coated fish fillet. All CGA/CS coating delayed increase in pH (*p* ≤ .05) and resulted brown color. However, only CS coating resulted in higher sensory scores (*p* ≤ .05) and controlled browning. Considering antioxidant properties and other quality parameters, CGA/CS coating might be applied commercially in fish preservation.

## Graphical abstract

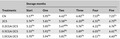

## 1 INTRODUCTION

With increasing fish consumption, problems related to fish preservation attracted the attention of consumers and researchers. Product‐specific storage needs to be developed for maintaining fish quality during storage. Snakehead fish (*Monopterus albus*) is a popular product and is loved by consumers in China. Its high content of water and protein results in easy deterioration, including changes in physicochemical properties, increasing microbial load, and decreasing nutritional and sensory qualities (Feng, Ng, Mikš‐Krajnik, & Yang, 2017a; Jääskeläinen et al., 2019; Luksiene & Buchovec, 2019). Several researches contributed to explore the nutrition, microbial safety, appearance, and product quality during storage (Chauhan et al., 2019; Feng et al., 2017a; Gokoglu, Yerlikaya, Topuz, & Buyukbenli, 2012; Kayim & Can, 2010; Sreelakshmi et al., 2019; Trabelsi et al., 2019). Fish processing and preservation have developed rapidly to provide new exciting knowledge for addressing industry requirements.

The application of edible coating with bioactive compounds in preservation has been successfully studied. The characteristics of edible coatings and their physicochemical nature have been given great interest (Fang, Lin, Warner, & Ha, 2018; Hassannejad, Nouri, Soltani, & Molavi, 2019). Macromolecules of protein, starch, modified starch, and polysaccharides have been applied in edible coating for preservation (Abdulkareem, Abdalsalam, & Bohan, 2019; Cardoso et al., 2019; Hassannejad et al., 2019). Chitosan (CS) coating is a nontoxic, attractive, and natural coating agent used in the food industry for inhibiting microorganism proliferation and lipid oxidization (Abdulkareem et al., 2019; Bharathi, Ranjithkumar, Chandarshekar, & Bhuvaneshwari, 2019; Reesha, Panda, Bindu, & Varghese, 2015). Use of additives in edible coating further enhances its activity in preservation by releasing antioxidants and antimicrobial substances (Ao et al., 2019; Cardoso et al., 2019). Thus, incorporation of chlorogenic acid (CGA) with chitosan coatings would exhibit oxygen barrier properties, since CGA has been known for its antioxidant activity (Gokoglu et al., 2012; Jiao, Wang, Yin, Xia, & Mei, 2018; Liu & Park, 2010). Regarding CGA incorporation, it is important to know the consequences of CGA/CS coating on qualities of snakehead fish fillets during cold storage. As the following exploration, CGA/CS coating is expected to maintain high quality of cool storage fish than CS coating.

Little research has been reported on CGA/CS coating in the preservation of fresh fish; thus, less information is available on the characteristics of CGA/CS‐coated fish. Therefore, a study was designed and carried out to evaluate the sensory qualities, texture, and color and to investigate oxidation of proteins and lipids of CGA/CS‐coated snakehead fish under vacuum package and stored at refrigeration temperature. This research will contribute to preserving fish and unveil the effects of CGA/CS edible coating on the product quality of fresh fish fillets during cold storage.

## 2 MATERIALS AND METHODS

### 2.1 Material and coating

Snakehead fish (15 cm long, 1.5 cm diameter, growth of 12 months) were purchased from Guangzhou Zhengyuan Food Technology Company Limited. Snakehead fish were cut into 3‐mm‐thick fillet (axial cutting). Chlorogenic acid was bought from Luye company in China; chitosan and other chemicals used were obtained from Nanjing Jiancheng Bioengineering Institute (Nanjing, China).

Coating solutions were prepared by putting chlorogenic acid (CID 5280633, B.R ≥ 0.98) into chitosan (CID 71853, deacetylation degree ≥ 90%, B.R ≥ 90%) solution (2%, w/w). Concentration of CGA was adjusted at 0%, 0.2%, 0.5%, and 1.0% (w/w) in 2% chitosan solution. Then, mixed solutions (CGA, CS, distilled water) with cosolvent of 1% citric acid were blended for 4 hr at 800 rpm using a magnetic stirrer for dissolution.

After fish fillets were soaked into the coating solutions for 30 s, the soaked samples were air‐dried at 40°C for 40 min under 1.8 m/s air velocity. A comparison had been performed by soaking in stilled water cosolvent of 1% citric acid for 30 s. Dried samples were placed on a glass tray (5 cm × 5 cm × 4 cm) with absorbing paper covered the bottom of the tray. Then, glass tray was packed at 0.7 MPa vacuum; the packed glass trays were stored at 2 ± 0.5°C in a refrigerator. Sampling and assay intervals were performed in every month for 5 months.

### 2.2 pH measurement

Snakehead fish were unwrapped, and pH was determined by a pH meter (SevenCompact S220‐Micro, Mettler Toledo Company). The pH of the samples was measured by inserting pH sensor into the fillet. When pH value reached maximum and was stable, it was documented with a precision of 0.01 (Cihlar, Drdlik, Cihlarova, & Hadraba, 2013).

### 2.3 Color measurement

CR400 colorimeter (Konica Minolta) was calibrated twice with a white board. After cutting the coating of samples, the sensor was placed on the surface of the sample and values of *L*, *a*, and *b* were measured and recorded. *L*, *a*, and *b* represent lightness, redness, and yellowness, respectively. Δ*E* was calculated using Equation (1) (Islam, Zhang, Adhikari, Xinfeng, & Xu, 2014).

(1) $$\Delta E=\sqrt{{\left(L_{1}-L_{0}\right)}^{2}+{\left(a_{1}-a_{0}\right)}^{2}+{\left(b_{1}-b_{0}\right)}^{2}}$$

In Equation (1), *L*_0_,* a*_0_,* b*_0_ and *L*_1_,* a*_1_,* b*_1_ represent the values of the fresh samples and stored samples, respectively.

### 2.4 Microbiological array

Total viable microbes were measured by incubation method (Fadıloğlu & Emir Çoban, 2018; Öz, 2018). Sample (20 g) was shifted into a sterilized stomacher bag (180 ml peptone water of 0.1 g/100 ml) and stomached in 2 min under 25°C. Concentration of samples was serially diluted in 10‐fold by injecting peptone solution of 0.1 g/100 ml, and diluted solutions (1 μl) inoculated and were spread on plate with MS medium (Murashige & Skoog, 1962). Inoculated plates were incubated at 37°C for 48 hr, and then, count of viable microbes was arrayed. Total viable microbes were calculated by multiplying dilution factor (fold) in log CFU/g.

### 2.5 Lipid oxidization

The thiobarbituric acid reactive substances (TBARS) were assayed with the earlier modified method for the lipid oxidization (Gokoglu et al., 2012; Öz, 2018; Özalp Özen, Eren, Pala, Özmen, & Soyer, 2011). Concisely, the fresh samples (15 g) were mixed with 30 ml trichloroacetic acid (TCA) (CID 6421) solution and homogenized at 7,000 rpm for 5 min. The homogenate was separated by centrifugation (5,478 *g*, 10 min), and liquid supernatant (5 ml) was shifted to 2‐thiobarbituric acid (5 ml, 20 mM) (CID 2723628). After agitation (800 rpm, 60 s), the liquid supernatant with 2‐thiobarbituric acid was incubated in 30 min under 90°C. The 532 nm absorbance was quantified by a spectrophotometer. A comparison was carried out using a blank sample. Blank solution consisted of 10% TCA and of 20 mM TBA (two solution, w/w = 1:1). Trichloroacetic acid solution consisted of TCA, ethylenediaminetetraacetic acid (CID 6049), and propyl gallate (CID 4947) (concentration, 10%, 0.1%, and 0.1%, respectively). Calibration unit was as mg malondialdehyde (MDA) (CID 10964) equivalent/kg sample (Fang et al., 2018).

### 2.6 Protein oxidization

Free thiol groups (protein oxidization) were arrayed with 5,5′‐dithiobis (2‐nitrobenzoic acid) (CID 11087) (Chauhan et al., 2019; Wang, He, Gan, & Li, 2018; Xu, Zhu, Liu, & Cheng, 2018). 2 g fish samples were homogenized with 30 ml 0.10 M tris buffer (CID 6503) (containing 5% SDS) in 2 min. The homogenates were water‐bathed at 80 ± 1°C for 30 min. And the homogenate was centrifugated (500 rpm, 15 min) for liquid supernatant. The concentration of 1.5 mg/ml centrifugated protein was adjusted by 5% SDS in 0.10 M tris buffer. 0.5 ml centrifugated protein was mingled with 10 mM DTNB and pH 8.0 tris (volume 0.5 ml, 2 ml, respectively). After incubation, the 412 nm absorbance was quantified by UV–spectrophotometer. A blank solution was arrayed using 0.10 M tris buffer (containing 5% SDS), pH 8.0 tris buffer, and 10 mM DTNB (0.5, 2, and 0.5 ml, respectively). Thiol group content was expressed by l‐cysteine (CID 5862) (standard substance) in nmol thiol/mg of protein (Fang et al., 2018; Sreelakshmi et al., 2019).

### 2.7 Texture analysis

Samples texture was analyzed by texture analyzer (TMS‐PRO, Food Technology Corporation). Test program was set as compressibility method (Peh, Khan, & Ch'Ng, 1999). Cylindrical probe (2 mm diameter) was used to penetrate through the fish fillets (thickness 3 mm). Pretest speed was 0.5 mm/s, test speed was 1 mm/s, and penetrate depth was 3 mm. Standard weight of 1.0 kg was used for calibration. Texture values were recorded, and the mean value was calculated.

### 2.8 Sensory evaluation

After removing package, samples (20 g) were fried in 170°C oil with some salt in 60 s. 20 trained panelists (10 men and 10 women, between 30 and 50 years) were recruited for sensory evaluation according to the earlier method (Xu, Song, et al., 2018). Fried samples (2 g) were randomly delivered to each panelist for evaluation. Evaluation scores were collected in different aspects of food. The score was analyzed by serial rank of 5, excellent; 4, good; 3, acceptable; 2, fair; and 1, unacceptable. Evaluation was operated in a panel test room at 25°C temperature under natural light.

### 2.9 Data analysis

Data were analyzed using analysis of variance (ANOVA), and mean comparisons were done using Duncan's multiple range test (DMRT) with a confidence level (*p* ≤ .05) of 95% using SPSS software (SPSS 20.0, IBM). All tests were carried out in triplicate unless stated. Data were presented as mean values with significant letters.

## 3 RESULTS AND DISCUSSION

### 3.1 pH value

Table 1 shows an upward trend in pH during storage of snakehead fish fillets. In noncoated fish fillets, the pH increased significantly from about 5.1 to 7.2 during storage, which significantly differed from the treated group (Table 1). The reason of high pH over the storage in control samples might be that volatile base nitrogen (TVB‐N) is formed by enzymatic hydrolysis of fish proteins (Chauhan et al., 2019; Trabelsi et al., 2019; Xu, Song, et al., 2018). This finding supported the fact that fresh fish is viable to decay. Higher pH values present higher content of TVB‐N formed by bacterial metabolites. In Table 1, characteristics of low pH of treated samples meant CS coating decreased fish albuminolysis. Chitosan coating barrier is approved for antimicrobial activity and suppresses bacterial growth again (Abdulkareem et al., 2019; Bharathi et al., 2019; Reesha et al., 2015). For 2CS, pH changes in coated samples increased markedly after 3 months, whereas for CN and 0.2 CGA/2CS the pH significantly increased after 2 and 4 months, respectively. On the other hand, for 0.5 CGA/2CS and 1.0 CGA/2CS the pH significantly increased after 5 months. These phenomena support high CGA content resulted in pH stability of snakehead fish during storage. It is implied that the additional CGA delayed increasing pH value of the samples. Simultaneously, chitosan coating was observed to be effective in suppressing product degradation during storage, which is in accordance with the earlier studies (Ao et al., 2019; Li, Wu, Wu, Yuan, & Hu, 2019; Luksiene & Buchovec, 2019; Olawuyi, Park, Lee, & Lee, 2019).

**Table 1:** pH trend of snakehead fish fillets subjected to different chlorogenic acid (CGA) chitosan coatings during storage

Treatments	Storage months					
	Start	One	Two	Three	Four	Five
CN	5.17^Aa^	5.95^Aa^	6.62^Cb^	6.82^Cb^	7.13^Bc^	7.23^Cc^
2CS	5.74^Aa^	5.81^Aa^	5.58^Aa^	6.20^Bb^	6.51^Bc^	6.75^Bc^
0.2CGA/2CS	5.22^Aa^	5.85^Aa^	5.67^ABa^	5.76^Aa^	6.22^Ab^	6.76^Bc^
0.5CGA/2CS	5.55^Aa^	5.92^Aa^	5.84^Ba^	5.89^Aa^	6.05^Aa^	6.61^Ab^
1.0CGA/2CS	5.70^Aa^	5.94^Aa^	5.81^Ba^	5.85^Aa^	6.11^Aa^	6.60^Ab^

Note

CN, noncoated; 2CS, 2% chitosan solution; 0.2CGA/2CS, 0.2% CGA (w/w) in 2% chitosan solution; 0.5CGA/2CS, 0.5% CGA (w/w) in 2% chitosan; solution; 1.0CGA/2CS, 1.0% CGA (w/w) in 2% chitosan solution. Data in the same column with different uppercase letter are significantly different, whereas data in the same row with different lowercase letter are significantly different (*p* ≤ .05).

### 3.2 Color evaluation

Table 2 presents the color qualities of the fish fillets during storage. Along with all the treated samples, *L* values decreased significantly with the storage time from 2 months of storage. The control samples had significantly (*p* ≤ .05) lower lightness than treated samples during the storage. Values (*a*) of all samples decreased significantly (*p* ≤ .05) during the 2 months (Table 2). This means that redness of samples decreased during storage. The reason might be the presence of brownness from oxidization of proteins and lipids during storage (Botsoglou, Christaki, Fletouris, Florou‐Paneri, & Spais, 2002; Cardoso et al., 2019; Chmiel, Roszko, Adamczak, Florowski, & Pietrzak, 2019; Sadeghinejad, Amini Sarteshnizi, Ahmadi Gavlighi, & Barzegar, 2019). *∆E* value in 2 or 5 months featured high level which meant big change in color. The reason was main contributor of fish lightness.

**Table 2:** Color trend of snakehead fish fillets subjected to different chlorogenic acid (CGA) chitosan coatings during storage

Color	Treatments	Storage months					
		Start	One	Two	Three	Four	Five
*L*	CN	49.77^Aa^	53.25^Aab^	46.15^Aab^	42.44^Ab^	40.52^Ab^	33.95^Ac^
	2CS	50.12^Aa^	55.95^Bb^	49.56^Bb^	47.33^Bbc^	43.33^Bc^	41.36^Bbc^
	0.2CGA/2CS	50.33^Aa^	55.55^Bb^	51.74^Bc^	48.15^Bc^	45.62^Cc^	42.65^Bc^
	0.5CGA/2CS	51.12^Aa^	55.25^Bb^	50.79^Bc^	48.77^Bc^	45.51^Cc^	42.71^Bc^
	1.0CGA/2CS	51.22^Aa^	55.35^Bb^	52.55^Bc^	48.97^Bc^	45.45^Cc^	42.21^Bc^
*a*	CN	2.56^Ad^	1.84^Ac^	1.51^Cb^	1.75^Cc^	1.72^Bc^	0.98^Ca^
	2CS	2.26^Ad^	1.38^Bc^	1.20^Ab^	1.23^Ab^	1.09^Ab^	0.85^Ba^
	0.2CGA/2CS	2.45^Ad^	1.44^Bc^	1.21^Ab^	1.22^Ab^	1.10^Ab^	0.83^Ba^
	0.5CGA/2CS	2.51^Ae^	1.51^Bd^	1.30^Bc^	1.53^Bd^	1.20^Bb^	0.77^Aa^
	1.0CGA/2CS	2.50^Ad^	1.45^Be^	1.33^Bb^	1.55^Bc^	1.34^Bb^	0.70^Aa^
*b*	CN	4.16^Aa^	4.10^Aa^	4.22^Aa^	4.21^Ba^	4.15^Aa^	4.12^Aa^
	2CS	4.23^Aa^	4.11^Ca^	4.14^Aa^	4.14^Aa^	4.11^Aa^	4.07^Aa^
	0.2CGA/2CS	4.33^Aa^	4.15^Ba^	4.12^Aa^	4.18^Aa^	4.02^Aa^	4.12^Aa^
	0.5CGA/2CS	4.15^Aa^	4.31^Ba^	4.32^Aa^	4.11^Aa^	4.07^Aa^	4.15^Aa^
	1.0CGA/2CS	4.14^Aa^	4.16^Ba^	4.00^Aa^	4.16^Aa^	4.05^Aa^	4.10^Aa^
Δ*E*	CN	–	3.55	3.76	7.37	9.28	15.89
	2CS	0.46	6.29	1.37	2.77	6.60	8.58
	0.2CGA/2CS	0.59	5.88	2.38	2.10	4.40	7.32
	0.5CGA/2CS	1.35	5.58	1.62	1.43	4.47	7.28
	1.0CGA/2CS	1.25	5.68	3.04	1.28	4.52	7.59

Note

CN, noncoated; 2CS, 2% chitosan solution; 0.2CGA/2CS, 0.2% CGA (w/w) in 2% chitosan solution; 0.5CGA/2CS, 0.5% CGA (w/w) in 2% chitosan; solution; 1.0CGA/2CS, 1.0% CGA (w/w) in 2% chitosan solution. Data in the same column with different uppercase letter are significantly different, whereas data in the same row with different lowercase letter are significantly different (*p* ≤ .05). *L*, *a*, and *b* represents lightness, redness, and yellowness, respectively.

Table 2 showed that the values of redness significantly differed among different treated profiles. Treated samples possessed lower redness value. This means CGA/CS treatments possess better color. After 2 months, high content of CGA resulted in upward trend of redness values. This trend meant the addition of CGA‐induced yellowness during storage (Table 2). During storage between 2 and 5 months, the redness values showed a similar pattern of change among chitosan coating and 0.2CGA/2CS treatments whereas 0.5CGA/2CS and 1.0CGA/2CS showed similar pattern of changing in redness values. The reason might be CGA oxidized to form yellow matter. In this work, *b* values represented slight fluctuation during the storage.

### 3.3 Total viable count

Total viable count value of snakehead fish fillet was subjected to the coating treatments during storage at 2°C (Table 3). After 5 months, increasing TVC values of about 6.5, 5.2, 5.3, 5.4, and 5.5 log CFU/g were responsible to noncoating sample and samples coated in 0.2CGA/2CS, 0.5CGA/2CS, and 1.0CGA/2CS. Naturally, 7 log CFU/g is the limit of microbiological safety in fresh fish fillets (Fadıloğlu & Emir Çoban, 2018; Fang et al., 2018; Olawuyi et al., 2019; Öz, 2018). In this study, coated samples were below 5.5 log CFU/g during storage at 2°C. In the absence of vacuum packaging, the shelf life of coated samples was within a week in refrigerator. Coating profiles combined with vacuum package met the demand of preservation of fresh fish fillets. It was implied that chitosan coating and vacuum package can inhibit the microbial growth (Table 3). Before 4 months, there was no difference between different chitosan‐treated profiles. These results suggest that CGA did not increase antimicrobial activity of chitosan‐coated samples to suppress microbial reproduction. It was also noticed that the TVC values of 2CS‐ and 0.2 CGA/2CS‐coated samples increased (*p* ≤ .05) until 3 months, while TVC values of 0.5CGA/2CS and 1.0CGA/2CS increased (*p* ≤ .05) until 4 months. This phenomenon indicates that coating of 0.5CGA/2CS and 1.0CGA/2CS delayed reaching maximum TVC value during storage, although no significant difference existed between different CGA content profiles (Table 3).

**Table 3:** Total viable microbes (TVC, CFU/g) of vacuum‐packaged snakehead fillets subjected different chlorogenic acid (CGA) chitosan coatings during storage

Treatments	Storage months					
	Start	One	Two	Three	Four	Five
CN	2.21^Aa^	4.55^Bb^	5.28^Bc^	6.05^Bd^	6.25^Bde^	6.55^Ce^
2CS	2.14^Aa^	3.34^Ab^	4.44^Ac^	4.64^Ac^	5.15^Ad^	5.25^Ad^
0.2CGA/2CS	2.10^Aa^	3.50^Ab^	4.60^Ac^	4.70^Ac^	5.11^Ad^	5.32^ABd^
0.5CGA/2CS	2.22^Aa^	3.52^Ab^	4.63^Ac^	4.95^Acd^	5.16^Ad^	5.45^Be^
1.0CGA/2CS	2.05^Aa^	3.51^Ab^	4.50^Ac^	4.74^Acd^	5.22^Ad^	5.51^Bd^

Note

CN, noncoated; 2CS, 2% chitosan solution; 0.2CGA/2CS, 0.2% CGA (w/w) in 2% chitosan solution; 0.5CGA/2CS, 0.5% CGA (w/w) in 2% chitosan solution; 1.0CGA/2CS, 1.0% CGA (w/w) in 2% chitosan solution. Data in the same column with different uppercase letter are significantly different, whereas data in the same row with different lowercase letter are significantly different (*p* ≤ .05); microbes' unit is log CFU/g.

### 3.4 Lipid oxidization

Table 4 shows the TBARS values of control and treated fish samples. The TBARS increased with the storage time in all samples. In control samples, TBARS values increased significantly (*p* ≤ .05) from 0.03 to 0.93 mg MDA/kg at 3 months, while the TBARS was found stable at 1.0 mg MDA/kg at the last 2 months. This increase in TBARS values implied severe oxidization in the control samples (Gokoglu et al., 2012; Öz, 2018). Compared with control samples, the TBARS of the coated samples increased slowly in the first 2 months (Table 4). 2CS‐treated samples showed significantly higher TBARS than 0.2CGA/2CS‐, 0.5CGA/2CS‐, and 1.0CGA/2CS‐treated samples after 1 month of storage. This clearly indicates that CGA delayed lipid oxidization process.

**Table 4:** Thiobarbituric acid reactive substances (TBARS, mg MDA/kg) of snakehead fish fillet subjected to different chlorogenic acid (CGA) chitosan coatings during storage

Treatments	Storage months					
	Start	One	Two	Three	Four	Five
CN	0.031^Aa^	0.195^Bb^	0.571^Cc^	0.935^Cd^	1.014^Cd^	1.124^Cd^
2CS	0.030^Aa^	0.111^Ab^	0.382^Bc^	0.552^Bd^	0.555^Bd^	0.571^Bd^
0.2CGA/2CS	0.044^Aa^	0.105^Ab^	0.277^Ac^	0.295^Ac^	0.333^Bd^	0.372^Ad^
0.5CGA/2CS	0.034^Aa^	0.097^Aa^	0.271^Ab^	0.285^Ab^	0.325^Bc^	0.337^Ac^
1.0CGA/2CS	0.037^Aa^	0.095^Aa^	0.209^Ab^	0.265^Ac^	0.272^Ac^	0.315^Ac^

Note

CN, noncoated; 2CS, 2% chitosan solution; 0.2CGA/2CS, 0.2% CGA (w/w) in 2% chitosan solution; 0.5CGA/2CS, 0.5% CGA (w/w) in 2% chitosan solution; 1.0CGA/2CS, 1.0% CGA (w/w) in 2% chitosan solution. Data in the same column with different uppercase letter are significantly different, whereas data in the same row with different lowercase letter are significantly different (*p* ≤ .05); TBARS unit is mg MDA/kg.

It has been reported that lipid oxidization in chitosan‐coated products impede by the chitosan macromolecules (Ao et al., 2019; Hassannejad et al., 2019; Pawlik et al., 2019), while slow release of CGA from edible coating also retards lipid oxidization(Jiao et al., 2018; Liu & Park, 2010). It can be seen from Table 4 that higher concentration of CGA resulted in lowest TBARS values during storage. Several researchers reported that additive antioxidant can effectively increase antioxidant properties of chitosan film (Feng et al., 2017a; Rui et al., 2017). Moreover, it has been reported that chitosan film with additional 1.5% cinnamon oil deters lipid oxidization in fish fillets (Ojagh, Rezaei, Razavi, & Hosseini, 2010). Here, this result shows that 0.5CGA/2CS‐ and 1.0CGA/2CS‐treated fish fillets implied low lipid oxidization from 2 to 5 months of storage (Table 4). Thus, the results of this experiment suggest that 0.5%‐1.0% CGA could be useful in the chitosan coating formula in preservation of snakehead fish.

### 3.5 Protein oxidization

Table 5 presents the values for free thiol group. The content of free thiol group values decreased significantly regardless of the treatments over the storage. After 5 months of storage, values dropped from about 79 nmol to 54 nmol, 50 nmol, 50 nmol, 47 nmol, and 40 nmol thiol/mg protein for the CN, 2CS, 0.2CGA/2CS, 0.5CGA/2CS, and 1.0CGA/2CS samples during storage, respectively. This indicates that oxidation increased with the storage time.

**Table 5:** Effect of chlorogenic acid/chitosan coating on protein oxidization (free thiol group values, nmol thiol/mg protein) of snakehead fish fillet

Treatments	Storage months					
	Start	One	Two	Three	Four	Five
CN	78.35^Ac^	74.82^Ac^	68.90^Bbc^	67.92^Cb^	59.11^Ca^	54.11^Ca^
2CS	79.51^Ac^	75.24^Ac^	65.52^Ab^	63.71^Ab^	55.21^Ba^	50.25^Ba^
0.2CGA/2CS	78.75^Ad^	76.33^Ad^	65.47^Ac^	62.15^ABc^	54.43^Bb^	50.51^Ba^
0.5CGA/2CS	77.35^Ad^	75.92^Ad^	65.26^Ac^	60.15^Ab^	50.44^Ba^	47.45^Ba^
1.0CGA/2CS	78.72^Ad^	75.42^Ad^	65.45^Ac^	57.15^Ab^	45.65^Aa^	40.71^Aa^

Note

CN, noncoated; 2CS, 2% chitosan solution; 0.2CGA/2CS, 0.2% CGA (w/w) in 2% chitosan solution; 0.5CGA/2CS, 0.5% CGA (w/w) in 2% chitosan solution; 1.0CGA/2CS, 1.0% CGA (w/w) in 2% chitosan solution. Data in the same column with different uppercase letter are significantly different, whereas data in the same row with different lowercase letter are significantly different (*p* ≤ .05).

There were no significant differences until 2 months of storage between the treatments. At third month, free thiol group values in 0.5CGA/2CS and 1.0CGA/2CS samples were significantly lower than 2CS and 0.2CGA/2CS. A lower value in 0.5CGA/2CS and 1.0CGA/2CS samples indicates higher oxidation, particularly in the 1.0% CGA/CS‐treated samples from 3 to 5 months. Interestingly, the control sample showed very less oxidation throughout the storage period.

The free thiol group values in 2CS‐ and 0.2CGA/2CS‐coated samples were lowered significantly (*p* ≤ .05) from third month to fourth month, while no significant changes were observed between fourth month and fifth month. This phenomenon represents that chitosan coating significantly lowered protein oxidization, and addition of 0.5% CGA or less did not affect much in free thiol group values (Jiao et al., 2018; Liu & Park, 2010), while higher amount of CGA did not improve the antioxidant properties of chitosan coating in snakehead fish during storage (Table 5).

The main reason of reduced protein oxidation is the application of chitosan coating and vacuum package which hindered product exposure to oxygen gas for responsible degradation (Gokoglu et al., 2012; Li et al., 2019; Özalp Özen et al., 2011). Hence, the antioxidant agent decreased oxygen gas to interact with fish fillets which inhibited the oxidization. Earlier studies have shown similar results that protein and lipid oxidization were delayed by adding extracts of plants (Gokoglu et al., 2012; Öz, 2018; Özalp Özen et al., 2011).

### 3.6 Texture analysis

Table 6 shows that hardness values change for CN, 2CS, 0.2CGA/2CS, 0.5CGA/2CS, and 1.0CGA/2CS samples, respectively, for 5 months of storage. In the first month, all coated samples showed no difference in hardness. The reason might be that chitosan formed into coating which presented no difference in hardness (Hassannejad et al., 2019; Jongberg, Terkelsen, Miklos, & Lund, 2014; Underwood et al., 2010). The other reason is that hardening of protein enhanced to high value (Table 6). This result is in accordance with the earlier finding (Fang et al., 2018).

**Table 6:** Hardness (N) of snakehead dish fillets subjected to different chlorogenic acid (CGA) chitosan coatings during storage

Treatments	Storage months					
	Start	One	Two	Three	Four	Five
CN	44.77^Aa^	43.54^Aa^	42.55^Aa^	39.52^Aa^	31.25^Bb^	29.15^Ab^
2CS	44.12^Aa^	42.54^Aa^	43.55^Aa^	32.33^Aa^	12.24^Ab^	15.22^Bb^
0.2CGA/2CS	44.55^Aa^	43.43^Aa^	48.32^Aa^	33.27^Aa^	13.47^Ab^	13.53^Bb^
0.5CGA/2CS	44.24^Aa^	44.34^Aa^	49.34^Aa^	38.22^Aa^	12.52^Ab^	12.37^Bb^
1.0CGA/2CS	44.71^Aa^	45.64^Aa^	45.14^Aa^	35.55^Aa^	17.59^Ab^	15.77^Bb^

Note

CN, noncoated; 2CS, 2% chitosan solution; 0.2CGA/2CS, 0.2% CGA (w/w) in 2% chitosan solution; 0.5CGA/2CS, 0.5% CGA (w/w) in 2% chitosan solution; 1.0CGA/2CS, 1.0% CGA (w/w) in 2% chitosan solution. Data in the same column with different uppercase letter are significantly different, whereas data in the same row with different lowercase letter are significantly different (*p* ≤ .05).

Different concentration of CGA also demonstrated similar hardness of all treated samples from 2 to 5 months. This meant additional CGA is not related to hardness. Synthetically, chitosan coating not only hindered the lipid oxidization but also impeded protein oxidization during fish fillet storage.

### 3.7 Sensory assessing

Table 7 shows sensory scores of samples subjected to treatments after 5 months under vacuum package at 2°C. Odors and texture exhibited no difference among coated samples whereas color and tastes demonstrated significant difference between different coating treatments. This was implied additional CGA increases preserving capability of chitosan coating and hence preserved better odor and color. High texture scores of samples existed using coating treatments, yet high taste scores occurred at 0.2CGA/2CS and 0.5CGA/2CS coating treatments. This result implied additional CGA enhances taste quality by deferring protein and fat oxidization as well as coating treatments enhanced textural quality. From average scores, all samples achieved above 3‐point excerpt for control samples. This means all treatments preserved fish quality in storage, compared with control samples. Considering tastes, addition of 0.2%–0.5% CGA was suggested for chitosan coating formula in fish storage.

**Table 7:** Sensory scores of the fried samples subjected to different chitosan coating after 5 months of storage

Parameters	Chlorogenic acid/chitosan coatings				
	CN	2CS	0.2CGA/2CS	0.5CGA/2CS	1.0CGA/2CS
Color	3.12^a^	4.22^b^	4.15^b^	3.52^a^	3.11^a^
Odor	2.21^a^	3.24^b^	3.32^b^	3.41^b^	3.34^b^
Taste	2.52^a^	2.55^a^	3.65^b^	3.57^b^	2.42^a^
Texture	3.21^a^	4.44^b^	4.65^b^	4.61^b^	4.50^b^
Average gradient	2.76^a^	3.61^b^	3.94^b^	3.78^b^	3.34^b^

Note

Different letters in the same row indicate significant differences (*p* ≤ .05); 5 = excellent, 4 = good, 3 = acceptable, 2 = fair, and 1 = unacceptable. CN, noncoated; 2CS, 2% chitosan solution; 0.2CGA/2CS, 0.2% CGA (w/w) in 2% chitosan solution; 0.5CGA/2CS, 0.5% CGA (w/w) in 2% chitosan solution; 1.0CGA/2CS, 1.0% CGA (w/w) in 2% chitosan solution.

## 4 CONCLUSIONS

Chitosan coating possesses antioxidant and antimicrobial properties in coating fish fillets; the additional CGA further enhanced antioxidant properties but not influence hardness of snakehead fish fillets in preservation. This work implied that CGA/CS coating will enhance the food safety and quality in preservation of fresh fish.

## CONFLICT OF INTEREST

The author(s) declared no potential conflicts of interest with the research, authorship, and publication of this article.

## ETHICAL APPROVAL

This study does not involve any human or animal testing.
